# Participation and retention in the breast cancer screening program in New Brunswick Canada

**DOI:** 10.1016/j.pmedr.2017.03.015

**Published:** 2017-03-23

**Authors:** James Ted McDonald, Yunli Wang, Zikuan Liu

**Affiliations:** University of New Brunswick, Fredericton, NB, Canada

**Keywords:** Breast cancer, Mammography, Socioeconomic status, Cancer screening, Administrative data

## Abstract

New Brunswick (NB) Canada uses its breast cancer screening service program to assess the extent to which eligible NB women are complying with mammography guidelines. While many studies have investigated factors associated with participation in periodic breast cancer screening in Canada and elsewhere, most work has relied on self-reported surveys or smaller scale primary data collection. Using a longitudinal administrative dataset for NB over the period 1996–2011 of 255,789 eligible women aged 45–69, this study examined demographic, socioeconomic and geographic factors associated with initial participation in regular screening at age 50 and ongoing retention in the program. Logistic regression was used to examine correlates of initial screening, while rescreening participation was estimated using survival analysis accounting for rescreening episodes. Initial screening participation was lower for women born outside of NB, many women living farther away from screening centers, women in rural areas, and higher for married women. In contrast, retention was higher for rural women and women recently arrived in NB. For both participation and retention, regional disparities across health zone persisted after controlling for observable personal and locational factors. The analysis highlights important characteristics to be targeted to increase screening but also that how health zones operate their screening programs exerts a very significant effect on the use of screening services by eligible women. This offers lessons for the design and evaluation of any cancer screening program.

## Highlights

• We analyze a linked longitudinal dataset on mammography screening from New Brunswick Canada.
• Screening at entry into the screening program at age 50 was lower for immigrants and rural residents.
• Rescreening conditional on previous screening was higher for rural residents and recent arrivals.
• Regional disparities in screening rates across health zone remained after controlling for covariates.

## 1 Introduction

Breast cancer is the most commonly diagnosed cancer among women and has the second highest mortality rate after lung cancer (Canadian Cancer Society's Advisory Committee on Cancer Statistics, 2016). Since the establishment of a breast cancer screening program in Canada beginning with British Columbia in 1988 and followed by the other Canadian provinces over the following ten years, breast cancer mortality rates have continued to decrease even while breast cancer incidence has fluctuated or stabilized (Canadian Cancer Society's Advisory Committee on Cancer Statistics, 2016). National guidelines in Canada recommend biennial mammography for asymptomatic women aged 50–69 and these guidelines have not changed for many years (Ringash, 2001, The Canadian Task Force on Preventive Health Care, 2011). Recent guidelines recommend biennial mammography for asymptomatic women aged 70–74 though based on weaker evidence (The Canadian Task Force on Preventive Health Care, 2011) so for continuity we focus on women aged 50–69 in this paper.

Participation and retention are two key dimensions for studies focusing on breast cancer screening and the degree of adherence of women to screening guidelines. The Public Health Agency of Canada defines participation as receiving a mammogram through an organized screening program and retention as returning for a mammogram through an organized screening program within 30 months of a previous mammogram (Canadian Partnership Against Cancer, 2015, Canadian Partnership Against Cancer, 2013). A recent performance report indicates that Canadian screening programs are not reaching target levels of 70% for overall participation nor the targets for retention following an initial screen (75%) or a subsequent screen (90%) (Canadian Partnership Against Cancer, 2015).

Participation studies investigate factors associated with utilization of breast cancer screening services, which include either initial screening or subsequent screening in a time period. Studies identified demographic (Zapka et al., 1989), geographical (Lerman et al., 1990), socio-economic factors (Zapka et al., 1989, Katz and Hofer, 1994, Maxwell et al., 2001) and psychological factors (Zapka et al., 1989, Lerman et al., 1990) contributing to variation in participation in breast cancer screening. For example, less education, minority ethnicities, etc. have been associated with lower participation in screening programs. Lower screening rates among Canadian women living in rural areas have been attributed to attitudinal differences than access to a screening center (McDonald and Sherman, 2010).

Earlier studies on participation included individuals who participated both in initial screening and subsequent screening. For most participants commencing involvement in a screening program at age 50, initial screening and retention with subsequent screening in accordance with guidelines would require at least eleven episodes of rescreening by age 69, assuming they remain asymptomatic. Thus there is considerable interest in considering the dynamics of the decisions to get (re)screened over the eligible age range. Conditional on initial screening, retention rates have been found to be relatively high for subsequent screening, in contrast to initial screening rates, and this is reflected in the higher screening targets for subsequent rescreening (Canadian Partnership Against Cancer, 2015, Canadian Partnership Against Cancer, 2013). Among those studies examining rescreening (Song and Fletcher, 1998, Sabogal et al., 2000, Barr et al., 2001, Rauscher et al., 2005, Bobo et al., 2004, Rosenberg et al., 2005, Calvocoressi et al., 2005, Fox et al., 2004), some have identified demographic (Song and Fletcher, 1998, Sabogal et al., 2000, Rauscher et al., 2005, Rosenberg et al., 2005, Fox et al., 2004), socioeconomic (Song and Fletcher, 1998, Sabogal et al., 2000), psychological factors (Rauscher et al., 2005, Fox et al., 2004), medical history (Bobo et al., 2004), and cost (Bobo et al., 2004) as correlated with rescreening.

Our study addresses a number of limitations in the existing literature. Many previous studies relied on self-reported data (Zapka et al., 1989, Lerman et al., 1990, Katz and Hofer, 1994, Maxwell et al., 2001, McDonald and Sherman, 2010, Tang et al., 2000) and examined initial rescreening only (Song and Fletcher, 1998, Barr et al., 2001, Bobo et al., 2004, Calvocoressi et al., 2005, Fox et al., 2004) or first and second follow-up (Rauscher et al., 2005, Bancej et al., 2005) only, without considering initial screening or retention over a longer period of time. Some existing studies have used administrative data on individuals participating in screening programs to study compliance with guidelines (Kiran et al., 2014, Corkum et al., 2014, Vigod et al., 2011) but use of Canadian administrative data has been limited (Bancej et al., 2005). To the best of our knowledge, no study in Canada has yet statistically investigated demographic, and socio-economic determinants of screening together, along with geographic factors such as travel distance to a screening center (see CPAC, 2014 (Canadian Partnership Against Cancer, 2014), for breast cancer screening rates decomposed by a range of income, demographic and geographic measures using survey and administrative data).

## 2 Materials and methods

We conduct an analysis of initial participation in screening and retention in the screening program using province-wide administrative data from multiple sources available over a 16 year period, 1996–2011. Screening data are linked at the individual level with cancer registry, resident data and citizen data on all women in the Province of New Brunswick Canada (NB) who were eligible for breast cancer screening through the provincial program. Almost all women are enrolled in the provincial health insurance system, with the only exceptions being certain women in the military or police and certain temporary residents to Canada.

### 2.1 Data sources

There are four main NB administrative data sources used for this study: Medicare Decision Support System (MDSS), breast cancer screening service (BCSS), Provincial Cancer Registry, and Vital Statistics data are all linked at the individual level by provincial Medicare number. MDSS yields population data for breast cancer screening by providing a record of the age, sex, language preference, Medicare insurance eligibility period, postal code and other demographic information on all NB residents who have been issued a Medicare card for provincially funded public health insurance.

The breast cancer screening program was introduced in NB in 1995 and extended across the province over the next year. The number of screening sites in NB did not change over the sample period nor were there any major changes to overall program organization once the program had been rolled out province-wide. While the number of administrative regional health authorities was reduced from 7 to 2 through amalgamation, the operation of the screening program remained at the level of the underlying health zones. For this reason we expect that there could be significant variation in screening uptake across zones.

The BCSS screening database provides a range of data on participants in the breast cancer screening program, including date and location of the screening, purpose of the screening, and information on participants such as education level. Women must have been screened at least once in order to be recorded in the BCSS screening database, so MDSS data allow women to be identified who are eligible for screening but never participated. Both BCSS and MDSS are longitudinal by design and linkable across individual and time.

Since a woman is eligible for enrollment in the screening program if she was not previously diagnosed with breast cancer, the NB Cancer Registry is used to identify any diagnosed cancer among women in the MDSS. Women with cancer diagnosed prior to age 50 are excluded from the linked sample, while a subsequent cancer diagnosis is treated as a right censored observation in the duration analysis. Similarly, women who died within the age range of eligibility for screening or who left the province are also treated as right censored. From BCSS, 131,591 subjects were extracted. From MDSS, records were extracted from all women aged 45–69 years between 1996 and 2011. In total, data on 255,789 women were extracted from MDSS.

The combined working dataset was assembled by the NB Department of Health and provided to the research team on the closed computer network of the NB Institute for Research, Data and Training on the UNB Fredericton campus. Ethics approval for this project was granted by the UNB Research Ethics Board.

### 2.2 Variables

The participation analysis focused on the initial participation decision of eligible women within 30 months of turning 50 in any year between 1996 and 2011. Demographic variables included marital status, preferred language (English or French), years living in NB and the previous place of residence if the individual previously moved to NB.

The Postal Code Conversion File was used to map individuals' postal codes to Statistics Canada geocodes for Dissemination Area (a neighborhood of 400–700 people). Urban/rural status is also derived from this procedure, which we categorize as: major cities (Moncton, Saint John, Fredericton), smaller cities and larger towns, and rural areas. To measure neighborhood SES, dissemination area was used to link to average household income in quartiles and % with a university degree from the Census of Canada 1996, 2001, and 2006. Other controls measured at the level of the woman's dissemination area included the % of French speakers as an indicator for a Francophone community, and % people using neither French nor English. Since these variables reflect area-level proportions, there are no obvious categorizations for these variables like there are for income quartiles. Thus we report results arising from a ten percentage point increase in the variable.

Determining travel distance is complicated by the fact that assigned screening facility is only observed for women who actually receive screening so must be imputed for those never screened. Imputation is not straight forward since a woman may not necessarily be assigned to her nearest screening site. Thus, for those women who received screening during the sample period, driving distance between residence postal codes and the postal code of the assigned screening site is calculated but for women who were never screened a screening center is assigned based on where a majority of women in the same postal code have gone for screening in that year. Driving distances are computed using ArcGIS and categorized into the following: 0–10 km, 10–16 km, 16–33 km, 33–50 km, over 50 km where cut-points are based on sample quintiles by distance.

Analysis of compliance with rescreening guidelines is restricted to women aged 50–69 who have been screened in the program at least once between the ages of 50 and 69. Restricting the sample to women in the BCSS allows us to use additional personal information on the participant: highest level of education obtained (< gr9, gr 9–12, some college, bachelor degree, higher degree) and region of birth (Canada, other developed English speaking countries, European countries, other). Two risk factors for breast cancer are included: full-term pregnancies (0, 1, 2, 3, 4 +) and age at first pregnancy (< 19, 19–24, 25–30, 31 +) based on the most recent value prior to when screening should have occurred. Variables by data source are listed in Table 1.

### 2.3 Statistical analysis

#### 2.3.1 Participation analysis

Participation is a binary outcome defined according to whether a given woman participated in the screening program within 30 months after turning 50. Although guidelines specify regular screening every 24 months for eligible women, a longer timeframe is used to allow for short delays in screening for reasons such as scheduling difficulties so as to avoid misclassification of intention to participate. Logistic regression was used to estimate the association between participation and the personal and neighborhood level characteristics of all eligible women after accounting for out-migration, mortality, or a previous diagnosis of cancer within 30 months of turning 50.

#### 2.3.2 Retention analysis

Survival analysis was used to examine the independent effect of various factors on the duration to rescreening following a previous screen. A conditional risk set model was used for survival analysis, where the data were set up as time-to-event when 2 + events occur for the same person. All participants in the screening program from 1996 to 2011 were considered, with the conditional risk set model stratified by event order to reflect first and subsequent rescreening as different outcomes. The assumption of this model is a subject is not at risk of a second event until the first event has occurred, and so on. Thus, the conditional risk set at time *t* for event *k* is made up of all subjects under observation at time t that have had event *k* − 1. Time to event is measured from the previous event and is not restricted to be a constant interval across individuals or over screening episodes. The last screening event before December 2011 of each woman was censored. Other censored observations included women diagnosed with breast cancer subsequent to an initial screening, women who left the province or who died, or women who turned 70. Using these results predictions of the probability of first or subsequent rescreening within 30 months of the previous screening can be generated and we present an illustration of these predictions in the results.

Other methods estimated included the Anderson-Gill survival model (Andersen and Gill, 1982), Poisson, pooled logistic and generalized estimating equation models (Advani et al., 2014). The main results are qualitatively similar to what is obtained from the other approaches.

## 3 Results

### 3.1 Descriptive statistics

**Fig. 1:**
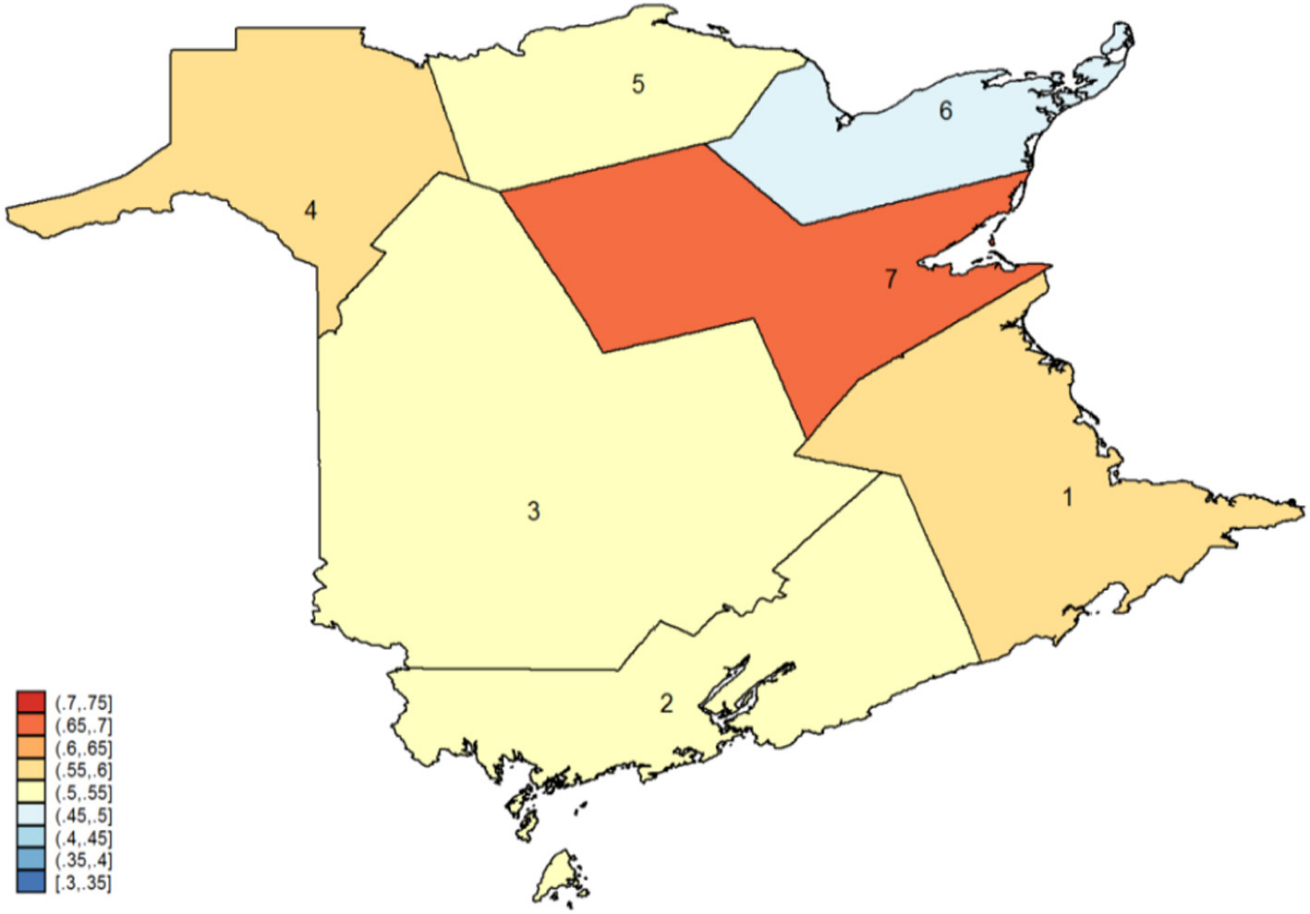
Biennial Participation Rate in NB for eligible women aged 50–69 by New Brunswick Health Zone in 2008–2009.

Fig. 1 presents overall biennial participation rates for all eligible women aged 50–69 by NB Health Zone in 2008–09, with cut-points based on 0.05 point intervals. Health Zones are administrative units within which certain health services such as cancer screening are organized and administered. These rates simply measure the proportion of eligible women who are screened within 30 months of a given year. It can be seen that participation rates for eligible women vary up to 25 percentage points across Zones, without accounting for differences in age and other factors. Table 2 reports the proportion of women who were screened within 30 months of turning 50, overall and by subcategory.

### 3.2 Multivariate model

Given that we have a large sample size and a fairly limited number of covariates, we include all relevant variables in the regression analysis. The results of the multivariate Logistic regression for initial screening at age 50 are reported in Table 3. The relationship between driving distance to assigned clinic and participation was non-monotonic, with moderate travel distances associated with lower participation but those needing to travel > 50 km having higher participation. Women living in areas with larger French speaking populations were more likely to participate in screening.

Notable again is the persistence of large differences in screening participation across zones. Participation rates in HR2, in HR4 and in HR6 remained lower than in the baseline HR1, while participation rates in HR5 and HR7 were significantly higher than baseline. At OR = 0.47 (CI 0.44–0.50), female residents of HR6 who turned 50 had less than half the odds of participating in screening as otherwise comparable women living in HR1, an adjacent health zone.

As an alternative, indicator variables were included for each of the 16 screening sites based on the site to which each woman was assigned. Results (not reported) indicate that significant variation is present across screening centers within health zones for those health zones with multiple screening centers. Estimated odds ratios range from 0.25 to 1.77, a range substantially wider than by zone.

### 3.3 Retention analysis

All subjects included in the retention analysis participated in the screening program at least once and were aged 50–69 in the screening years 1996–2011, which included 381,470 observations of screening by 112,575 individuals. Since the sample is conditional on women being in the screening database, some additional variables available in BCSS were also included as described above.

Results from the conditional risk set model are shown in Table 4. The hazard rate ratio (HRR) is the relative risk of returning for rescreening following a previous screen so an estimated HRR > 1 indicates a higher likelihood of being rescreened (a shorter duration until rescreening). Demographic factors indicate that women who are relatively younger, married, and who have a higher level of education are statistically associated with a shorter duration of time until rescreening. Women from other English speaking countries and Europe are as likely to return for rescreening than Canadian born women but women from other regions are less likely (HRR 0.91, CI 0.84–1.00). Women with > 3 children were less likely to be rescreened while women older than age 25 at first birth were more likely. Women living in NB < 11 years are more likely to return than women born in NB or living there for > 10 years, in contrast to the results for participation in screening upon turning age 50.

Geographic factors in retention analysis revealed different patterns than in the participation analysis. A reduced likelihood of returning for rescreening was observed when travel distances to clinics were longer than 15 km, although rescreening was more likely for women residing in rural areas. For statistics based on area of residence, a higher percentage of university graduates in an area is associated with a shorter duration to rescreening. As well, living in an area with a higher concentration of French speakers as well as speakers of another language was associated with a longer duration to rescreening. The result for French language is in contrast to what was observed for initial participation in screening. Year effects (not reported) indicate no significant pattern over time except for the first two years when the program scaled up.

**Fig. 2:**
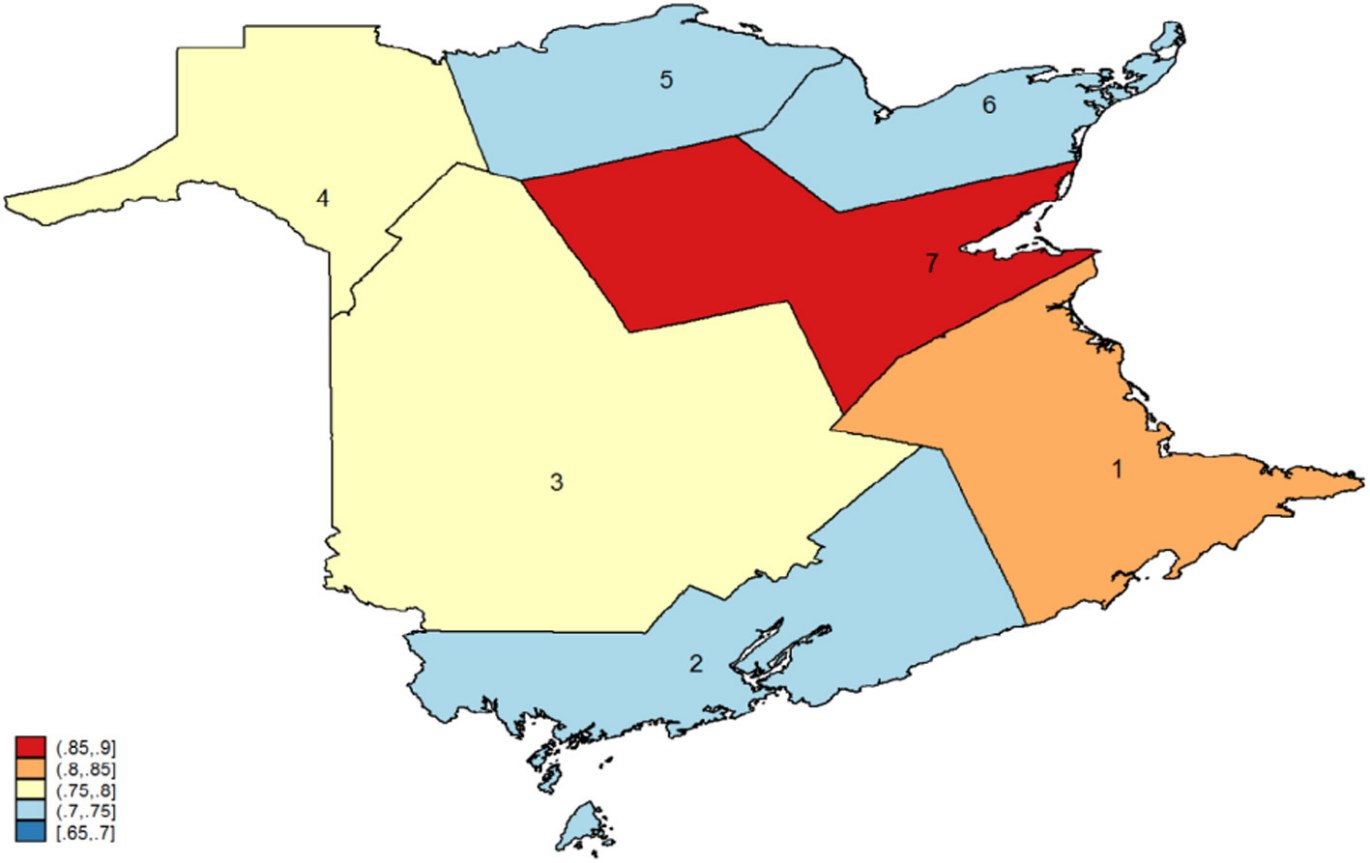
Predicted probability of first rescreening within 30 months of initial screening at age 50 by health region in 2009.

The results reinforce the importance of regional differences in terms of rescreening as well as initial participation. Women living in health zones 4 and 7 are more likely to return than women in region 1, while women in zones 2, 5 and 6 are less likely to return than region 1. Results for zones 4 and 5 are the opposite of what was estimated for participation. To illustrate what the estimates imply for the probability of rescreening within 30 months of a particular episode of screening, we present predicted probabilities by health zone for first rescreening within thirty months after initial participation in the screening program, computed at mean values for the estimates. Predicted probabilities by health region for the ‘average’ woman are less than but close to the target of 75% for three of the seven zones (Canadian Partnership Against Cancer, 2015) and that there remain large differences in rescreening rates across health zones (Fig. 2).

## 4 Discussion

Identifying disparities in participation and retention in screening programs is critical for developing effective screening services and improving care delivery. Our study was the first Canadian study examining both participation and retention for breast cancer screening programs using administrative data. Linking to population data allowed examination of the prevalence of NB women who never participated in mammographic breast cancer screening.

Participation and retention in the screening program are distinct behaviors, are based on different samples, require different methodologies and so should be analyzed separately. Results of the multivariate Logistic regression for initial screening agreed with findings from other studies (Canadian Partnership Against Cancer, 2013, Zapka et al., 1989, Lerman et al., 1990, Katz and Hofer, 1994, Maxwell et al., 2001, McDonald and Sherman, 2010, Tang et al., 2000, Song and Fletcher, 1998, Sabogal et al., 2000, Barr et al., 2001, Rauscher et al., 2005, Bobo et al., 2004, Rosenberg et al., 2005, Calvocoressi et al., 2005, Fox et al., 2004). Results indicated certain demographic, geographic, and socioeconomic factors were significant determinants of both initial participation and retention but not always in the same direction. More recent arrivals to NB were significantly less likely to participate in breast cancer screening upon turning 50, but for those recently arrived women who did participate, they were also more likely to continue to comply with the guidelines after joining the program. One apparently anomalous result is urban/rural status. Women living in medium cities or rural areas were less likely to have participated in screening upon turning 50 than women in urban areas, but for women who did participate initially they were also more likely to get rescreened. Similarly, while initial participation was positively related to income quartile, rescreening was not. These results might reflect a form of sample selection where by virtue of having participated in screening at least once, such women are demonstrating their commitment to periodic screening.

Of note for health system planners is that the importance of geographic variables identified in an early study (Maxwell et al., 2001) are also important in NB. In terms of travel distance, with the exception of initial participation by women living 50 km + from an assigned screening center, longer travel distances were associated with both lower participation and lower retention in the program. Longer travel distances may constitute a barrier for women to continue to participate in regular mammography screening. The result for initial participation among women 50 km + from their assigned screening center may also be due to unobserved regional effects correlated with travel distance, or may reflect assignment errors in imputing screening clinic for those who were never screened. Further analysis indicates that this is not an artefact of how screening is assigned. Unlike what was found in recent work in the US (Alford-Teaster et al., 2016), a non-trivial number of women chose to participate in screening facilities that were not close to their geographically nearest facility. Related to this, there remained unexplained quantitatively significant differences across health zones in both participation and retention.

To examine this in more depth, we replaced the indicators for health zones with indicators for particular screening facility (noting that most zones have multiple screening sites), the disparity in participation and rescreening across facilities became even larger. Furthermore there were observed differences across screening centers within particular health zones. These results suggest that how health zones and screening centers are operating their screening programs are exerting a significant effect on the use of screening. Each health zone operates the screening program in the region, sending reminder letters, booking appointments, managing wait times and operating/maintaining local equipment, while particular screening facilities may differ by the language of regular use, wait times, and staff/equipment characteristics. Screening rates could be brought closer to recommended levels with dissemination of tracking and follow-up methods from the more successful zones/facilities to zones with lower retention in the program. It may be that where women live is as important for screening as what their characteristics are, suggesting that the patient experience and the organization of service delivery are key. Specific aspects of how screening programs are operated are unfortunately not regularly collected by the health regions and so are not considered in the current work but will be an important avenue for future work.

Although administrative data are able to provide information for the whole NB population of eligible women, they have some limitations. Most of the missing data was processed as a separate category in the statistical analysis. For women who were never screened, estimation used assigned screening facility based on other women in the same postal code who were participating in screening. Postal code was the key variable used to generate geographical variables and link social economic variables but may represent geographically broad areas, especially in rural NB.

## Conflict of interest

The authors have no conflicts of interest to declare.

## Figures and Tables

**Table 1:** Variables by Data Source.

Medicare decision support system (*n* = 255,789 women aged 45–69)
• Age
• Postal code of residence
• Language preference
• Marital status
• Years resident in NB
Breast cancer screening system (*n* = 131,591 women aged 45–69)
• Education level
• Date of screening
• Location of screening
• Parity, age at first birth
Vital statistics
• Date of death
Cancer registry
• Date and site of diagnosed cancer
Area-level census information
• Income quartile
• Proportion of adults with a university degree
• Proportion of adults using French as main language at home
• Proportion of adults using neither English nor French as main language at home

**Table 2:** Proportion of eligible women by category who were screened within 30 months of NB women turning 50 1996–2011.

Variable	Proportion screened
All	0.55
Marital status	
Married	0.59
Div/sep/wid	0.50
Single	0.42
% missing = 0%	
Preferred language	
English	0.56
French	0.50
% missing = 5%	
Urban/rural	
City (Moncton, Saint John, Fredericton)	0.58
Medium city	0.54
Rural	0.50
% missing = 1%	
Distance	
0–10 km	0.56
10–16 km	0.56
16–33 km	0.56
33–50 km	0.54
Over 50 km	0.54
% missing = 1%	
Health region	
HR1	0.60
HR2	0.54
HR3	0.58
HR4	0.51
HR5	0.60
HR6	0.43
HR7	0.61
% missing = 1%	
Average household income	
Lowest quartile	0.50
Second lowest quartile	0.53
Second highest quartile	0.56
Highest quartile	0.60
% missing = 3%	
N	91,917

**Table 3:** Factors associated with participation in screening program within 30 months after NB women turning 50, 1996–2011 (*n* = 91,917).

Variable names	Odds ratio	95% CI
Marital status		
Married	1	–
Single	0.52	(0.50–0.54)
Other	0.69	(0.67–0.72)
Preferred Language		
English	1	–
French	1.04	(0.99–1.08)
Years in NB		
50 years	1	–
1–10 years	0.72	(0.68–0.76)
11–50 years	0.86	(0.80–0.92)
Urban/rural		
Moncton, Saint John, Fredericton	1	–
Smaller city	0.91	(0.87–0.95)
Rural	0.83	(0.79–0.87)
Distance		
0–10 km	1	–
10–16 km	1.04	(0.99–1.10)
16–33 km	0.93	(0.90–0.97)
33–50 km	0.88	(0.84–0.92)
Over 50 km	1.10	(1.04–1.15)
Health region		
HR1(Moncton)	1	–
HR2	0.81	(0.78–0.85)
HR3	1.02	(0.97–1.07)
HR4	0.62	(0.58–0.67)
HR5	1.09	(1.01–1.18)
HR6	0.47	(0.44–0.50)
HR7	1.15	(1.07–1.23)
Area-level characteristics		
Average household income		
Quartile 1	1	–
Quartile 2	1.07	(1.02–1.12)
Quartile 3	1.15	(1.10–1.20)
Quartile 4	1.27	(1.21–1.33)
Proportion of adults with a degree	2.77	(1.91–4.02)
Proportion with French home language	1.31	(1.23–1.39)
Proportion with nonofficial home language	0.31	(0.19–0.51)

**Table 4:** Conditional risk model estimates of the time until rescreening, NB women aged 50–69 screened at least once,1996–2011 (*n* = 100,020).

Variable names	Hazard rate ratio	95% CI
Age		
50–54	1	–
55–59	0.80	(0.79–0.81)
60–64	0.78	(0.77–0.79)
65–69	0.85	(0.84–0.87)
Early screening	1.26	(1.24–1.27)
Education		
Grade 9 or less	1	–
Some high school	1.01	(0.99–1.02)
High school diploma	1.04	(1.02–1.05)
Some college/university	1.04	(1.02–1.06)
University degree	1.04	(1.02–1.06)
Marital Status		
Married	1	–
Single	0.99	(0.97–1.01)
Other	0.99	(0.98–1.00)
Preferred language		
English	1	–
French	0.98	(0.97–1.00)
Parity		
2	1	–
0 or 1	1.01	(0.99–1.02)
3	0.99	(0.98–1.00)
Over 3	0.99	(0.97–1.00)
First Birth		
19–24	1	–
Under 19	0.99	(0.98–1.00)
25–30	1.02	(1.00–1.03)
Over 30	1.02	(1.00–1.04)
Years in NB		
Born in NB	1	–
1–10 years	1.18	(1.16–1.20)
11–50 years	1.01	(0.99–1.04)
Birth Region		
Canada	1	–
English countries	0.99	(0.94–1.05)
Other European countries	0.98	(0.87–1.16)
Others	0.91	(0.84–0.99)
City or rural		
City (Moncton, Saint John, Fredericton)	1	–
Medium city	1.04	(1.03–1.05)
Rural	1.09	(1.07–1.11)
Distance		
0–10 km	1	–
10–16 km	1.00	(0.99–1.02)
16–33 km	0.98	(0.97–0.99)
33–50 km	0.97	(0.95–0.98)
Over 50 km	0.95	(0.94–0.97)
Health region		
HR1(Moncton)	1	–
HR2	0.90	(0.87–0.91)
HR3	0.94	(0.92–0.95)
HR4	1.03	(1.01–1.05)
HR5	0.85	(0.83–0.86)
HR6	0.87	(0.85–0.89)
HR7	1.21	(1.19–1.24)
Dissemination area characteristics		
Percentage with university degree	1.30	(1.17–1.44)
Average household income		
Quartile 1	1	–
Quartile 2	0.99	(0.98–1.01)
Quartile 3	0.99	(0.98–1.00)
Quartile 4	1.00	(0.98–1.01)
Percentage with French home language	0.97	(0.96–0.99)
Percentage with nonofficial home language	0.85	(0.72–1.00)

## References

[bb0130] Advani P.S., Ying J., Theriault R. (2014). Ethnic disparities in adherence to breast cancer survivorship surveillance care. Cancer.

[bb0135] Alford-Teaster J., Lange J., Hubbard R. (2016). Is the closest facility the one actually used? An assessment of travel time estimation based on mammography facilities. Int. J. Health Geogr..

[bb0125] Andersen P.K., Gill R.D. (1982). Cox's regression model for counting processes: a large sample study. Ann. Stat..

[bb0070] Barr J.K., Franks A.L., Lee N.C., Herther P., Schachter M. (2001). Factors associated with continued participation in mammography screening. Prev. Med..

[bb0080] Bobo J.K., Shapiro J.A., Schulman J., Wolters C.L. (2004). On-schedule mammography rescreening in the national breast and cervical cancer early detection program, caner epidemiology. Biomarkers & Prevention.

[bb0100] Bancej C.M., Maxwell C.J., Onysko J., Eliasziw M. (2005). Mammography utilization in Canadian women aged 50 to 69: identification of factors that predict initiation and adherence. Can. J. Public Health.

[bb0090] Calvocoressi L., Stolar M., Kasl S.V. (2005). Applying recursive partitioning to a prospective study of factors associated with adherence to mammography screening guidelines. Am. J. Epidemiol..

[bb0025] Canadian Partnership Against Cancer (2013). Report from the Evaluation Indicators Working Group: Guidelines for Monitoring Breast Cancer Screening Program Performance.

[bb0140] Canadian Partnership Against Cancer (2014). Examining Disparities in Cancer Control: A System Performance Special Focus Report.

[bb0020] Canadian Partnership Against Cancer (2015). Breast Cancer Screening in Canada: Monitoring and Evaluation of Quality Indicators - Results Report, January 2009–December 2010.

[bb0005] Canadian Cancer Society's Advisory Committee on Cancer Statistics (2016). Canadian Cancer Statistics.

[bb0115] Corkum M., Urquhart R., Kephart G., Hayden J.A., Porter G. (2014). Breast and cervical cancer screening behaviours among colorectal cancer survivors in Nova Scotia. Curr. Oncol..

[bb0095] Fox P., Arnsberger P., Owens D. (2004). Patient and clinical site factors associated with rescreening behavior among older multiethnic, low-income women. The Gerontologist.

[bb0040] Katz S.J., Hofer T.P. (1994). Socioeconomic disparities in preventive care persist despite universal coverage: breast and cervical cancer screening in Ontario and the United States. J. Am. Med. Assoc..

[bb0110] Kiran T., Wilton A.S., Moineddin R., Paszat L., Glazier R.H. (2014). Effect of payment incentives on cancer screening in Ontario primary care. Ann. Fam. Med..

[bb0035] Lerman C., Rimer B., Trock B. (1990). Factors associated with repeat adherence to breast cancer screening. Prev. Med..

[bb0045] Maxwell C., Bancej C., Snider J. (2001). Predictors of mammography use among Canadian women aged 50–69: findings from the 1996/97 National Population Health Survey. Can. Med. Assoc. J..

[bb0050] McDonald J.T., Sherman A. (2010). Determinants of mammography usage across rural and urban regions of Canada. Can. J. Rural Med..

[bb0075] Rauscher G.H., Hawley S.T., Earp J.A.L. (2005). Baseline predictors of initiation vs. maintenance of regular mammography use among rural women. Prev. Med..

[bb0010] Ringash J. (2001). Canadian task force on preventive health care. Preventive health care, 2001 update: screening mammography among women aged 40–49 years at average risk of breast cancer. Can. Med. Assoc. J..

[bb0085] Rosenberg L., Wise L.A., Palmer J.R. (2005). A multilevel study of socioeconomic predictors of regular mammography use among African-American women, cancer epidemiology. Biomarkers & Prevention.

[bb0065] Sabogal F., Merrill S.S., Packel L. (2000). Mammography rescreening among older California women. Health Care Financ. Rev..

[bb0060] Song L., Fletcher R. (1998). Breast cancer rescreening in low-income women. Am. J. Prev. Med..

[bb0055] Tang T.S., Solomon L.J., McCracken L.M. (2000). Cultural barriers to mammography, clinical breast exam, and breast self-exam among Chinese-American women 60 and older. Prev. Med..

[bb0015] The Canadian Task Force on Preventive Health Care (2011). Recommendations on screening for breast cancer in average-risk women aged 40–74 years. Can. Med. Assoc. J..

[bb0120] Vigod S.N., Kurdyak P.A., Stewart D.E., Gnam W.H., Goering P.N. (2011). Depressive symptoms as a determinant of breast and cervical cancer screening in women: a population-based study in Ontario, Canada. Arch. Womens Ment. Health.

[bb0030] Zapka J.G., Stoddard A.M., Costanza M.E., Greene H.L. (1989). Breast cancer screening by mammography: utilization and associated factors. Am. J. Public Health.

